# Negative impact of online gambling problematic in disabled and non-disabled university students: exploring the risk profile

**DOI:** 10.3389/fpsyg.2024.1429122

**Published:** 2024-09-03

**Authors:** Raquel Suriá-Martínez, Fernando García-Castillo, Esther Villegas-Castrillo, Carmen López-Sánchez, Carmen Carretón-Ballester

**Affiliations:** ^1^Department of Communication and Social Psychology, Universidad de Alicante, Alicante, Comunidad Valeciana, Spain; ^2^Department of Educatión, Universidad de Alicante, Alicante, Comunidad Valeciana, Spain; ^3^Department of Social Work and Social Affairs, Universidad de Alicante, Alicante, Comunidad Valeciana, Spain

**Keywords:** online gambling problematice, university students, disability, intervention, program

## Abstract

**Introduction:**

The rise of online gambling has brought about significant concerns, particularly regarding its impact on university students. This issue becomes even more complex when considering students with disabilities.

**Objectives:**

This research examines the gambling profile and beliefs of university students based on whether they have a disability. In turn, it seeks to identify if there is a typology of at-risk gamblers according to the disability variable. Finally, it aims to find out the prevalence of gambling among students depending on whether they have a disability and the typology of at-risk gambler.

**Method:**

A total of 704 university students (135 with disabilities and 569 without disabilities) completed the NODS Belief Questionnaire to assess problems associated with gambling and to generate a typological grouping of risk gamblers, as well as a questionnaire designed specifically for the gambler profile.

**Results:**

It was found that a small percentage of participants engage in gambling on a daily basis, with a higher frequency among students with disabilities. In addition, it was observed that the behavior and concern about financial expenditure on gambling interferes with their daily activities and is a cause for concern, with higher risk being observed to a greater extent in students with disabilities.

**Discussion:**

These data suggest the importance for universities and relevant authorities to address these problems comprehensively, providing adequate resources for students with disabilities and promoting a culture of wellbeing that discourages problematic gambling activities and encourages healthy alternatives for entertainment and stress management.

## 1 Introduction

In today's digital age, online entertainment has become an integral aspect of young people's lives. From the rise of social media to the explosion of online video games, today's young people are immersed in a virtual world that offers a wide range of entertainment activities.

In reference to leisure preferences, empirical evidence reveals that online games, and especially new forms of gambling, such as online poker, online casino games (blackjack, roulette, and online slots) and sports betting (games legalized by Law 13/2011 on the regulation of gambling by the Ministry of Finance and Public Administrations of Spain (2011), are the most attractive and in demand by the youth population (Labrador et al., 2021).

However, despite the demand and availability of games, like any technological tool, their excessive misuse can lead to various personal and social problems (Beranuy Fargues et al., 2009; Zboralski et al., 2009; Echeburúa and de Corral, 2010; Esparza-Reig et al., 2023; Jitoku et al., 2024).

Thus, it is not new news that the number of young people whose activities and social life are solely web-based is increasing every day, and that the number of hours they spend in front of the computer is growing at the same rate, taking time away from other activities they used to do (Krishnamurthy and Chetlapalli, 2015; González-Bueso et al., 2018; Aydin and Kuş, 2023).

In reviewing the literature on this topic, most authors have focused on socio-demographic variables such as age (Beranuy et al., 2009; Labrador and Villadongos, 2010; Auer and Griffiths, 2023) or gender (Muñoz-Rivas et al., 2003; Sánchez-Martínez and Otero, 2009; Esparza-Reig et al., 2023) as possible risk factors for the problematice of gambling and betting offered over the Internet.

In terms of age and the problematicive use of games available on the Internet, it has become a challenge for health professionals, being more and more frequent among young people (Liu and Potenza, 2007; Greer et al., 2023). In fact, it is increasingly prevalent among the university population, as this group has the highest percentage of problematic and problematicive Internet users (Krishnamurthy and Chetlapalli, 2015; Osuna et al., 2021). University students become potential users because the internet is one of the main tools in academic activities, which enables and justifies online time; in addition, their study schedules and academic tasks are very flexible, and there is little adult supervision over their online activities. Add to this the fact that youth is a time of increased vulnerability for the development of addictions, because cognitive control and risk perception are not fully developed and therefore ineffective (Casey et al., 2005), and because of difficulties in setting limits (Liu and Potenza, 2007; Greer et al., 2023), and a high vulnerability to Internet use and, possibly, to gambling and betting will be favored (Kuss et al., 2013). These data are evidenced by different prevalence studies conducted with this population, which indicate that a significant number of university students present internet addiction in some form, with percentages ranging from 3.2% (Kuss et al., 2013) to 11% (Shao et al., 2018).

With regard to gender and the risk of online gambling problematice, current prevalence figures according to the study by Tristán et al. (2020) indicate that 63.5% of men and 56.9% of women aged 15–64 years gamble with money on the internet. With special mention of the particularly worrying fact that the prevalence of the disorder among them has decreased by 1% for men and increased by 0.2% for women in 2020.

Other related variables that empirical evidence has highlighted and that may influence a person's greater vulnerability to excessive or problematicive use of the Internet and, in particular, online gambling and betting, are certain personality characteristics. These include introversion, low self-esteem, high level of sensation seeking, impulsivity, inadequate coping with problems, etc. (Widyanto and Griffiths, 2006; Mehroof and Griffiths, 2010; Duplaga and Szulc, 2019). Deficits in interpersonal relationships also increase psychological vulnerability to addictive gaming behavior (Muñoz-Rivas et al., 2003; Echeburúa and de Corral, 2010; Balanzá-Martínez et al., 2020; Blasco et al., 2021).

Similarly, certain physical conditions such as stigmatizing illnesses (Høybye et al., 2005) and disability (Finn, 1999) could influence the problematicive use of the internet (Finn, 1999; Høybye et al., 2005; Bringué and Sádaba-Chalezquer, 2008; Krishnamurthy and Chetlapalli, 2015; Osuna et al., 2021), as well as the gambling platforms available online.

In the disability context, the ease of access to a myriad of sites and resources without the constraints of having to travel, and thus being able to share their interests, concerns or needs, allows people with physical limitations to make changes in their lives that might otherwise be difficult or even impossible (Finn, 1999). This, as different researchers indicate, has been evidenced in different areas of life such as the academic (Alcantud et al., 2000), the social (Suriá, 2015), or the work context (Cuervo and Menéndez, 2005), and of course, in recreation (Suriá, 2015), and leisure and gambling through the internet (García-Ruíz and Bonilla-del-Río, 2020).

Although most often in individuals with a diagnosis of mental illness (schizophrenia, bipolar disorder, personality disorder, etc.), pathological gambling has also been detected in those with physical, sensory and intellectual disabilities. However, based on the published literature on the population of youth with disabilities and online gambling, very few studies have been published on the use of these sites and the problematic effects of problematice associated with gambling among people living with this disability (Solish et al., 2010; McCormack et al., 2013; Pallesen et al., 2021; Pitt et al., 2021). Moreover, no studies have been conducted in our country.

Regarding online gambling and students with disabilities, the subject of this paper, access to online content logically facilitates access to online gambling platforms and has brought gambling into the privacy of our pockets; a place where gambling behavior is nourished until recreational gambling becomes a disorder. It is at this point that the biopsychosocial damage to the individual can no longer be hidden (Balanzá-Martínez et al., 2020; Fazeli et al., 2020; Blasco et al., 2021).

Despite the repercussions of overuse in virtual sites, it is easily verifiable that we are bombarded by online gambling and betting agencies across different media platforms. It is unlikely that any internet user will not stumble across several banners or eye-catching links inviting them to gamble, regardless of their status or age. This is especially true for people, as well as for those affected by limiting conditions such as having a disability (Solish et al., 2010; Mateu-Mateu and Navarro-Gómez, 2015; Suriá, 2015; Osuna et al., 2021; Pitt et al., 2021).

Notwithstanding the existence of a gap in the literature on the problematice or addiction of people with disabilities to online gambling and betting, this research is based on the need to focus on the population of students with disabilities and the use of online gambling for two fundamental reasons. First, given the vulnerability of university students to excessive use of these games, we believe that it may be very relevant to focus attention on the group of students who participate in this type of online leisure. Second the availability and access that people with disabilities find online provides them with an open window to the world, making these virtual spaces more attractive. Hence, it is plausible to think that the students with the handicap of living with disabilities are particularly likely to spend more time online, and therefore to engage in addictive behavior on the Internet, and possibly online gambling and betting.

In this university context, it should be borne in mind that it is a context of training, development and biopsychosocial integration. In order to achieve the comprehensive achievement of young people, it becomes the task of universities to generate an environment that contributes to the wellbeing and personal development of their students (García-Ruiz and Escoda, 2021). Accordingly, the transition to the university stage demands a higher level of autonomy, responsibility, and achievement, which is why this educational cycle can become a strengthening scenario for the growth of personal and psychosocial health or a problematic space that generates risk behaviors.

On the basis of these considerations, the objectives of this research are 4-fold:

1. First, to examine the gambling profile, i.e., gambling type preference, as well as the frequency of participation in online gambling and betting among university students according to whether they have a disability.
2. The second objective aims to explore whether there are differences in beliefs and concerns between disabled and non-disabled university students who gamble and/or bet on the Internet.
3. The third objective is to identify whether there is a typology of at-risk gamblers based on the disability variable.
4. Finally, the fourth objective aims to find out the prevalence of gambling among these students depending on whether they have a disability and the type of at-risk gamblers.

## 2 Method

### 2.1 Participants

The analyses in the present study are based on data from a sample of university students (*N* = 704) from 25 public and private universities in Spain, recruited through convenience sampling and an open online questionnaire between January and March 2024. Females represent 24.9% (*n* = 175) of the sample and males 75.1% (*n* = 529); ages range from 18 to 44 years with 14.1% between 18 and 22 years, 38.2% between 23 and 27 years, 26% between 28 and 33 years, 21.7% between 34 and 38 years, and 10.5% 39 from 44 years; 93.5% of the sample are undergraduate students: 31.1% of year 1, 14.3% of year 2, 12.6% of year 3, 35.4% of year 4, and 6.5% postgraduate and/or masters students and 18.5% (*n* = 130) of the sample indicate having some kind of recognized disability: 8.7% motor, 3.7% mental, 3.0% hearing, 1.7% cognitive, and 1.4% visual (Table 1).

**Table 1:** Sociodemografic profile.

**Sociodemographic profile**		***N***	**%**
Age	18–22	99	14.1
	23–27	269	38.2
	28–33	183	26.0
	34–38	81	11.5
	39–44	72	10.2
Sex	Male	529	75.1
	Female	175	24.9
Disability	Non-disability	569	80.8
	Disability	135	19.2
Undergraduate	1°	219	31.1
	2°	101	14.3
	3°	89	12.6
	4°	249	35.4
	Máster o Doctorado	46	6.5
	Total	704	100.0

### 2.2 Instruments

*Student's socio-demographic data collection form (gender, age, and if disabled or not)*.

*Questionnaire aimed at the profile of use, preferences, and money spent on gambling and betting* (typology of gambling preferences, frequency, and expenditure on gambling participation).

*NODS Belief Questionnaire*, DSM-IV Screen for Gambling Problems (Gerstein et al., 1999), as adapted from Becoña (2004). To assess the problems associated with gambling and to generate a typological grouping of at-risk gamblers (third objective), an adaptation of five variables called “beliefs and concerns” from the NODS (Gerstein et al., 1999) was used. The NODS has been developed with the aim of providing a reliable assessment instrument for pathological gambling that meets DSM-IV criteria. It consists of 17 items covering the 10 DSM-IV criteria for pathological gambling. The cut-off point is 5 or more criteria for probable pathological gambling. It is currently, together with the SOGS, the most widely used instrument in studies of pathological gambling. The 5-item subscale was used to assess beliefs and concerns about gambling and betting participation. Cut-off point > 1. Scores are distributed into four profiles according to risk: no risk (0); at risk (1 or 2); problem (3 or 4); pathological (5). To obtain the diagnostic criteria for gambling disorder based on the DSM-5, the sample is classified into four categories according to their level of problem gambling: non-risk gambler, at-risk gambler, problem gambler and pathological gambler.

For testing whether the adaptation of the questionnaire had adequate psychometric properties, the five variables included in the “beliefs” dimension were measured on a four-grade scale: 1 = never, 2 = sometimes, 3 = most of the time, and 4 = always. The reliability analysis of the scales measuring the variables by means of Cronbach's alpha is adequate (Cronbach's alpha = 0.783). To ascertain and validate the factorial structure of the scale, an Exploratory Factor Analysis was carried out using the Principal Component Analysis and by means of Varimax rotation, obtaining acceptable results: Kaiser-Meyer-Olkin (KMO) Index = 0.817; Bartlett's Test of Sphericity = 1.021 (*df* = 10) (*p* < 0.01). Finally, a single factor explains 54.8% of the variance in the data.

### 2.3 Procedure

The data collection procedure consisted of applying the questionnaire to the sample of students. The sample was selected between January and February 2024, with the prior informed consent of the participants in the study. Access was relatively starightforward; the sample came from university degrees at the University of Alicante. The questionnaire was hosted on Google and through the collaboration of the Vice-Rectorate for Research was advertised on campus for dissemination. The estimated application time was ~10 min.

### 2.4 Data analysis

Non-parametric tests were used to examine statistically significant differences between the groups (with and without disability). Thus, for the first objective, to analyse the differences between the students in the variable type of game, frequency, and type of at-risk gambler, the chi-square homogeneity test was used. For the second objective, to examine whether there are statistically significant differences in the “beliefs” dimension, depending on whether the students have a disability, the Mann-Whitney *U* test was employed. For the achievement of the third objective, to identify whether there is a typology of at-risk gambler according to the disability variable, the chi-square test (*X*^2^) was applied. Finally, the Kruskal-Wallis *H*-test was used to determine the prevalence of gambling and expenditure (fourth objective). Subsequently, *post-hoc* comparisons were performed using the Games-Howell *Post-Hoc* Test for median gambling expenditure by gambler type and disability.

### 2.5 Design

In selecting university students grouped according to the presence or absence of disability, a cross-sectional design was used, with a purposive and non-probabilistic approach. This type of design can be useful for studying specific groups in particular contexts, such as universities.

## 3 Results

**Figure 1:**
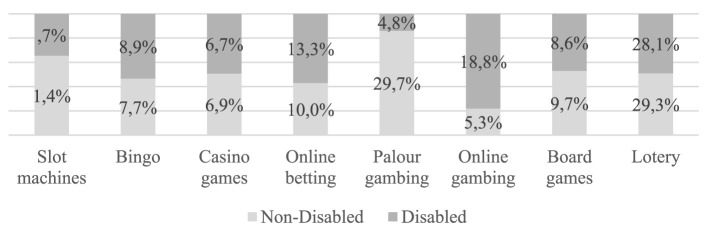
Game of chance preference.

In reference to the preference in the type of games, 74.1% of the students surveyed indicated that they usually play offline gambling and board games, while the remaining 25.9% prefer online gambling and betting. When looking at gambling preferences according to disability and non-disability (Figure 1), the results showed statistically significant differences between students with and without disabilities, albeit with a small effect, only in online games: students with disabilities ($X_{13}^{2}$ = 12.198, *p* < 0.005, *Phi* = 0.191) show a higher prevalence for online gambling (18.8%) and online betting ($X_{13}^{2}$ = 8.146, *p* < 0.005, *Phi* = 0.162) than those without disability (5.3%), while students without disability ($X_{13}^{2}$ = 4.898, *p* < 0.005, *Phi* = 0.096) show a higher prevalence for parlor betting (29.7%) compared to participants with disability (4.8%).

In terms of frequency of participation, 23.9% of the sample of students usually gamble once a year, compared to 76.1% who have a prevalence of gambling in the last 30 days. When examining the higher or lower frequency among gamblers (Table 2), it was observed that 49.7% of the players have low prevalence (once a month), 16.2% moderate prevalence (once a week), 4.7% moderate-high prevalence (several times a week), and 2.6% high prevalence (every day, several times a day).

**Table 2:** Frequency of participation based on having or not having a disability.

**Frequency**		**Non-disabled**	**Disabled**	**Total**
No freq.	Once a year	142 (25.0%)	26 (19.3%)	168 (23.9%)
Low freq.	Once a month	290 (51.0%)	60 (44.4%)	350 (49.7%)
Moderate freq.	Once a week	88 (15.5%)	26 (19.3%)	114 (16.2%)
Moderate high freq.	Several times a week	25 (4.4%)	8 (5.9%)	33 (4.7%)
Moderate/high freq.	Every day	14 (2.5%)	7 (5.2%)	21 (3.0%)
High freq.	Several times a day	10 (1.8%)	8 (5.9%)	18 (2.6%)
	Total	569 (100%)	135 (100%)	704 (100%)
	*X* ^2^	14.065	*p* < 0.05	
	Phi	1.41	*p* < 0.05	

^**^p ≤ 0.01, ^*^p ≤ 0.05.

Focusing the results on the prevalences of preoccupying gambling based on having or not having a disability, the analyses showed that at moderate-high (3.0%), and high (2.6%) frequency, statistically significant differences were observed. Students with a disability (${\text{X}}_{4}^{2}$ = 14.06, *p* < 0.005, *Phi* = 1.41) indicated higher moderate-high (5.2%) and high (5.9%) prevalence for gambling than their non-disabled peers at both moderately high (2.5%) and high (1.8%) prevalence (Table 2).

Regarding the second objective (Table 3), and after exploring the scores of the five variables of the “beliefs or concerns” dimension included in the NODS questionnaire, which form a 4-point Likert-type scale (1 = never, 2 = sometimes, 3 = most of the time, and 4 = always), the analysis of the median^1^ (*Mdn*) of the five variables is around “Sometimes” (*Mdn* = 2; Range = 3) and the analysis of the percentage distribution of the most extreme responses of the scale (3 = most of the time and 4 = always) reveals that 39.9% of the sample of students indicated items 3 and/or 4 as a minimum on any of the five variables: *gambled to win back what they lost* (10.5%), *felt bad about spending money gambling* (15.2%, *d* = 0.40), *gambled more than they intended to* (21.1%), *borrowed or stole money to gamble or pay back debts* (7.8%, *d* = 0.30), and *missed class or family/personal plans to gamble or bet* (15.4%). When examining disability and non-disability scores, the results showed that, in two of them, statistically significant differences with a small-medium effect size were observed, reflecting that students with disabilities tend to a higher degree to *feel bad about spending money gambling* (PR = 407.4; ∑% i3/i4 = 32.3%), than non-disabled participants (PR = 340.1; ∑% i3/i4 = 11.3%), although they tend to *ask for or steal money to gamble or pay back debts* to a lesser extent (PR = 310.8; ∑% i3/i4 = 0.8%) than non-disabled students (PR = 361.9; ∑% i3/i4 = 9.4%).

**Table 3:** Analysis of the items “beliefs and concerns” and disability.

**Sometimes**	***Mdn*/range**	**Disability**	
		***U***	***d***
Gambled to win back what they lost	2.0 (3)	36,679	-
Felt bad about spending money gambling	2.0 (3)	30,165^*^	0.40
Gambled more than they intended to	2.0 (3)	35,863	-
Asked for/stole money to gamble or pay back your debts	2.0 (3)	31,892^*^	0.30
Missed school/family/personal plans due to gambling or betting	2.0 (3)	37,061	-

Effect size (d): 0.2 small, 0.5 medium, 0.8 large; (f): 0.1 small, 0.25 medium, 0.4 large.

^**^p ≤ 0.01.

^*^p ≤ 0.05.

As far as the identification of the typology of at-risk gamblers is concerned, a high percentage are at risk of developing gambling problems, ~4.7% already have problems and 5.6% have an existing addiction (Table 4). Thus, with regard to the typology of at-risk gamblers according to whether they have a disability, the analyses indicated the existence of statistically significant differences, although in all analyses the effect is medium (*Phi* ≤ 0.20), showing a higher percentage of students with disabilities in the problematic profiles. In this way, a profile of pathological (11.1%) and at-risk gamblers (5.9%) was observed ($X_{17}^{2}$ = 14.035, *p* < 0.05, *Phi* = 1.141).

**Table 4:** Test of homogeneity (*X*^2^), at-risk gambler type based on having or not having disability.

	**Non-disabled**		**Disabled**		**Total**	
No risk	432	75.1	86	63.7	518	76.3
At risk	88	15.5	26	19.3	114	23.9
With problems	25	4.4	8	5.9	33	4.7
Pathological	24	4.3	15	11.1	39	5.6
Total	569		135		704	
*X* ^2^	14.035^*^		*p* < 0.05			
Phi	1.41^*^		*p* < 0.05			

^**^p ≤ 0.01, ^*^p ≤ 0.05.

Regarding the gambling prevalence and expenditure (Table 5), the median expenditure^2^ (*Mdn* = 18 €; Range = 120) by students who gamble on the different online games is <20 € and as the level of risk increases the expenditure increases, reaching 67.5 € among students with problems or 45 € among pathological gamblers. The Kruskal-Wallis H-test determines that there are statistically significant differences between the types of gamblers with and without disabilities with respect to expenditure (*H* = 81.655, *df* = 7, *p* = 0.001). *Post*-*hoc* analyses using the Games-Howell statistic revealed that, in most significant pairwise combinations, students with disabilities spend more than students without disabilities and increase this expenditure as their level of risk increases: students with disability and not at risk had higher expenditure (*Mdn* = 45 €) than students without disability and not at risk (*Mdn* = 18 €, *p* = 0.025) 95% CI (2.21, 55.79), students with disability and at risk had higher expenditure (*Mdn* = 73 €) than students without disability and at risk (*Mdn* = 18 €, *p* = 0.001) 95% CI (15.97, 55.58) and disabled and at-risk students, the stratum with the highest observed expenditure, spent more (*Mdn* = 105.5 €) than students without disability and with problems (*Mdn* = 45 €, *p* = 0.050) 95% CI (−1.73, 74.17).

**Table 5:** Games-Howell *post-hoc* test for median expenditure on games by gamblers typology and disability.

						**95% CI**	
**1/** ***n***	**2/** ***n***	***Mdn*** €**/Av. range (1)**	***Mdn*** €**/Av. range (2)**	**Error deviation**	***p***	**Lower limit**	**Upper limit**
With dis., no risk/ *n* = 43	No dis., no risk/ *n* =322	45 (424.45)	18 (309.22)	8.457	0.025	2.21	55.79
With dis., at risk/ *n* = 73	No dis., at risk/ *n* = 207	90 (474.05)	18 (324.44)	6.409	0.001	15.97	55.58
With dis., with problems/*n* = 12	No dis., with problems/ *n* = 207	105.5 (585.00)	45 (455.36)	11.757	0.050	−1.73	74.17
With dis., pathological/*n* = 2	No dis., no risk/ *n* =322	90 (553.50)	18 (309.22)	2.146	0.001	53.87	66.97
	With dis., no risk/ *n* = 43	90 (553.50)	45 (424.45)	8.180	0.009	5.34	57.50
	No dis., at risk/ *n* = 207	90 (553.50)	18 (324.44)	2.839	0.000	49.00	66.38
	With dis., at risk/ *n* = 73	90 (553.50)	90 (474.05)	5.746	0.007	3.98	39.86
	No dis., with problems/*n* = 207	90 (553.50)	45 (455.36)	8.347	0.034	1.34	55.11

^**^p ≤ 0.01, ^*^p ≤ 0.05.

## 4 Discussion

Nowadays, the increasing prevalence of online gambling among university students has raised serious concerns about the potential risks involved. This phenomenon is rooted in the context of a generation that has witnessed an explosion in the availability and accessibility of gambling, facilitated by the legalization of gambling activities and technological advances that have resulted in a wide range of gambling options via the internet and mobile devices. Thus, the increased supply of legalized gambling and the proliferation of online platforms have created an environment conducive to young people's participation in these activities. The extensive culture of gambling and online betting in Spain can be explained primarily by the fact that, in 2011, Spain passed legislation regulating online betting, creating a clear and secure legal framework for operators and players. Additionally, the high level of internet penetration and the widespread use of mobile devices in Spain have facilitated access to online betting platforms. This has made it easy and convenient for users to participate in gambling from anywhere and at any time. Furthermore, advertising campaigns, sports sponsorships, and promotional offers have contributed to increasing the visibility and social acceptance of online gambling. As a result, gambling and betting are often seen as a normalized and popular form of entertainment and leisure among much of the Spanish population. However, for certain groups, such as university students with disabilities, participation in gambling may be increased due to the inherent characteristics of the disability.

Therefore, the aim of this study is to test whether disability may be a risk factor for online gambling participation. To achieve this goal, four specific objectives have been identified: first, to examine the type of games and frequency of participation in gambling among students with and without disabilities; second, to explore the beliefs and concerns related to gambling among students with disabilities; third, to research whether there is a profile of at-risk gamblers among students with disabilities; finally, to conduct a comparative analysis of the prevalence of gambling and associated expenditure among university students, based on their disability status.

Foremost, the results of the first objective indicate that, in general, university students tend to play offline games, although not very often. However, there are statistically significant differences in the type of gambling when the disability variable is taken into account, with disabled students preferring online gambling, while non-disabled students prefer gaming in arcades.

As some authors indicate, participation in online gambling among students with disabilities may be due to a variety of reasons. Aspects such as accessibility to connect with friends and engage in leisure activities without the physical limitations they might face in other social settings may be a justifiable reason for participation in this type of entertainment (Gómez Hernández, 2000; Pallesen et al., 2021). In this sense, this group may see their degree of autonomy and independence reduced, limiting their attendance at leisure and recreational venues, which, in some way, may be conditioned by architectural barriers. The same applies to certain mass events such as concerts or festivals that involve crowds, or the need to depend on other companions for transport (Pitt et al., 2021). Finally, possible mobility, visual or hearing impairments may reduce autonomy and condition travel, sports or outdoor activities (Emerson et al., 2021).

Likewise, disability can reduce face-to-face interpersonal interaction and lead to problems of loneliness, especially in the juvenile stage, which is characterized by the need for personal affirmation through peer recognition (Luque-Parra et al., 2014). These circumstances can lead to demotivation or a tendency to engage in more passive leisure practices, in which direct or face-to-face participation is not necessary as in other types of gambling (Solish et al., 2010; Pitt et al., 2021).

In terms of time spent, ~25% of students showed a moderate, moderately high and high frequency of participation. Thus, when looking at the disability variable, comparisons reveal that students with disabilities showed a higher frequency of participation, with a moderately high and high frequency (16.9%), which are higher percentages than those observed in the data for students without disabilities (8.7%).

In relation to these findings, different studies have shown that internet gambling can be particularly attractive to many vulnerable groups, such as those who spend long periods of time at home. This may be the case of students with disabilities who, due to their circumstances, may spend more time alone at home and therefore have more opportunities to go online and opt for recreational opportunities such as these (Corney and Davis, 2010; Suriá, 2012; McCormack et al., 2013; Osuna et al., 2021).

In this regard, several authors note that online games have emerged among people as a form of entertainment and socialization, and can provide students with disabilities a way to connect with friends and engage in playful activities without the physical limitations they may encounter in other social settings (Pallesen et al., 2021; Martínez et al., 2024; Suriá et al., 2024). At the same time, online platforms can level the playing field for people with disabilities, providing opportunities for participation in recreational activities that might be more challenging in physical settings. Finally, online gambling can serve as a way to escape the realities of their disability and the limitations they are confronted with in everyday life, providing a temporary distraction from their physical or emotional challenges. This can lead to an ideal leisure context that can be accessed for recreational activities that do not involve added effort, such as the leisure-oriented spaces available on a multitude of websites (Morahan-Martin and Schumacher, 2000; McCormack et al., 2013; Duplaga and Szulc, 2019).

In reference to their beliefs about gambling and betting (second objective), the results reflect that, to some extent, they have spent more than they intended to, felt bad about gambling, or missed school or family appointments because of gambling. This, as indicated by the DSM-V, denotes a certain danger or risk of addiction, since their behavior and preoccupation with the financial cost of gambling begins to interfere with their daily activities and becomes a cause for concern, with more discomfort being observed to a greater extent in students with disabilities.

Possibly, if most students have not yet emancipated themselves from their families, those with disabilities are less likely to do so. Similarly, if labor market integration is difficult for people, it is much more difficult for people with disabilities to access the labor market (Suriá and Ortigosa Quiles, 2022; García et al., 2023). Therefore, in most of the cases of these students, the way of earning an income depends on their family members, so they do not have a high income. This and the fact that they tend to gamble at home (in which family members and/or carers live together very often) may generate more worries or concerns than in students without disabilities.

In this respect, and after reviewing the scarce literature on Internet addiction in the population of students with disabilities, the results of the study coincide with other authors. For example, Suriá (2015) focused on the comparative analysis of Internet problematice in a sample of university students with and without disabilities. The results reflected the existence of a certain inclination among disabled people to use this resource for online social and recreational leisure, as well as its access as an entertainment and leisure strategy. Moreover, although no apparent changes were obtained that affected the daily lives of these young people in their day-to-day activities, the findings did show that it generated some kind of discomfort or dependence if they did not go online. The results concluded that people with disabilities, who are regular users of the net as a form of leisure, recognized some negative consequences in their lives.

This hypothesis is strengthened when examining the last objective that analyses the profile of the at-risk gambler and the money invested in the games, and the results show that as the level of at-risk gambler increases, so does the amount spent. And as mentioned above, this is more pronounced in the case of students with disabilities, who spend more than non-disabled students.

Although the published literature on disabled gamblers and online gambling is sparse, some exceptions have focused on the relationship between the two variables. For example, Pitt et al. (2021) studied the benefits and negative effects of gambling and other risky behaviors in a group of young Australians with disabilities. The results showed that, although participants were cautious about alcohol consumption, most had gambled, and some of them indicated that they had had problems with online gambling.

Another international study conducted by McCormack et al. (2013) examined the characteristics and predictors of online gambling for problem vs. non-problem gamblers. Their results showed that the most addicted gamblers were more likely to be young, male, smokers, engage in these activities alone and have a disability.

Finally, in another European study by Pallesen et al. (2021), they analyzed changes over time and identified predictors of online gambling among three representative Norwegian samples over a 6-year period (2013–2019). Consistent predictors of online gambling were students, male gender, being unemployed and having a disability.

In summary, online gambling addiction is a serious problem that can affect people of all ages and backgrounds, including students with disabilities. People with disabilities may face unique challenges that can increase their vulnerability to such addictions. For example, they may turn to online gambling and betting as a way of escaping the difficulties they experience in everyday life, or they may be attracted by the accessibility and ease of access offered by online platforms.

Despite the relevance of the present work, the study on online gambling and betting problematice among students, both with and without disabilities, may encounter several limitations that could affect the validity and generalizability of the results of a study with this population and methodology. First, the sample size. A study with only 704 participants may not be representative of the general university student population. The sample may be too small to detect small effects or significant differences. Also, generalizability. Since the study is limited to university students with and without disabilities, the findings may not be generalizable to other age groups, educational levels or cultural backgrounds. Regarding the questionnaire and response bias, the results of the questionnaire may be biased by participants' tendency to respond in a socially desirable way or to exaggerate or minimize their online gambling addiction problems. The self-selection of the sample should also not be overlooked. Students who agree to participate in the study may have different characteristics than those who choose not to participate, which could bias the results. Finally, there is the lack of diversity. The sample may lack diversity in terms of types of disability, severity of disability, gender, ethnicity, or other important factors that could influence online gambling addiction.

To address these limitations, it would be useful to conduct a study with a larger and more diverse sample. It would also be helpful to use multiple data collection methods (e.g., qualitative interviews in addition to questionnaires) and ensure that measurement instruments are adapted to address the specific needs of people with disabilities. By proactively addressing these issues and providing the necessary support, it is possible to help prevent problem gambling and promote the wellbeing of all students, including those with disabilities. It is important to consider the additional risk factors associated with disability when designing prevention and treatment strategies for gambling addiction in this population group. Students with disabilities may require specialized access to resources and support to address needs related to gambling addiction, highlighting the importance of including mental health and wellness services specifically tailored to their needs. This would allow for the establishment of policies that promote responsible gambling and the creation of prevention tools for vulnerable students.

## Data availability statement

The raw data supporting the conclusions of this article will be made available by the authors, without undue reservation.

## Ethics statement

The studies involving humans were approved by Comité de Ética de la Universidad de Alicante. The studies were conducted in accordance with the local legislation and institutional requirements. The participants provided their written informed consent to participate in this study. Written informed consent was obtained from the individual(s) for the publication of any potentially identifiable images or data included in this article.

## Author contributions

RS-M: Conceptualization, Data curation, Formal analysis, Investigation, Methodology, Supervision, Validation, Visualization, Writing – original draft, Writing – review & editing. FG-C: Investigation, Supervision, Visualization, Writing – original draft, Writing – review & editing. EV-C: Conceptualization, Formal analysis, Methodology, Visualization, Writing – original draft, Writing – review & editing. CL-S: Formal analysis, Investigation, Methodology, Visualization, Writing – original draft, Writing – review & editing. CC-B: Conceptualization, Formal analysis, Methodology, Visualization, Writing – original draft, Writing – review & editing.

## Footnotes

^1^The Kolmogorov-Smirnov test corroborates the alternative hypothesis of non-normality for all five variables.

^2^The Kolmogorov-Smirnov test (Stat. = 0.280; df = 704; *p* = 0.001) corroborates the alternative hypothesis of non-normality of the variable “Game expenditure.”
